# Epidemiology of Foodborne Norovirus Outbreaks, United States, 2001–2008

**DOI:** 10.3201/eid1810.120833

**Published:** 2012-10

**Authors:** Aron J. Hall, Valerie G. Eisenbart, Amy Lehman Etingüe, L. Hannah Gould, Ben A. Lopman, Umesh D. Parashar

**Affiliations:** Centers for Disease Control and Prevention, Atlanta, Georgia, USA (A.J. Hall, V.G. Eisenbart, A. Lehman Etingüe, L.H. Gould, B.A. Lopman, U.D. Parashar);; University of Illinois, Urbana, Illinois, USA (V.G. Eisenbart);; and North Carolina State University, Raleigh, North Carolina, USA (A. Lehman Etingüe)

## Abstract

In the United States, the leading cause of foodborne illness is norovirus; an average of 1 foodborne norovirus outbreak is reported every day. The more we know about how this virus is spread and in which foods, the better we can ward off future outbreaks. A recent study identified the most common sources of foodborne norovirus outbreaks as ready-to-eat foods that contain fresh produce and mollusks that are eaten raw, such as oysters. Most implicated foods had been prepared in restaurants, delicatessens, and other commercial settings and were most often contaminated by an infected food worker. Although possible contamination during production, harvesting, or processing cannot be overlooked, food safety during meal preparation should be emphasized. Food handlers should wash their hands, avoid bare-handed contact with ready-to-eat foods, and not work when they are sick.

## Abstract 2

Keywords: norovirus, foodborne disease, outbreaks, attribution, United States, viruses

## Medscape CME Activity

Medscape, LLC is pleased to provide online continuing medical education (CME) for this journal article, allowing clinicians the opportunity to earn CME credit.

This activity has been planned and implemented in accordance with the Essential Areas and policies of the Accreditation Council for Continuing Medical Education through the joint sponsorship of Medscape, LLC and Emerging Infectious Diseases. Medscape, LLC is accredited by the ACCME to provide continuing medical education for physicians.

Medscape, LLC designates this Journal-based CME activity for a maximum of 1 *AMA PRA Category 1 Credit(s)*^TM^. Physicians should claim only the credit commensurate with the extent of their participation in the activity.

All other clinicians completing this activity will be issued a certificate of participation. To participate in this journal CME activity: (1) review the learning objectives and author disclosures; (2) study the education content; (3) take the post-test with a 70% minimum passing score and complete the evaluation at www.medscape.org/journal/eid; (4) view/print certificate.

**Release date: September 17, 2012; Expiration date: September 17, 2013**

## Learning Objectives

Upon completion of this activity, participants will be able to:

- Describe general characteristics and outcomes of US norovirus outbreaks, based on an analysis of data reported during 2001-2008 to the CDC Foodborne Disease Outbreak Surveillance System
- Describe sources of US norovirus outbreaks, based on an analysis of data reported during 2001-2008 to the CDC Foodborne Disease Outbreak Surveillance System
- Describe recommended interventions to reduce the frequency and effects of foodborne norovirus outbreaks, based on an analysis of data reported during 2001-2008 to the CDC Foodborne Disease Outbreak Surveillance System

## CME Editor

**Carol E. Snarey, MA**, Technical Writer/Editor, *Emerging Infectious Diseases. Disclosure: Carol E. Snarey, MA, has disclosed no relevant financial relationships.*

## CME Author

**Laurie Barclay, MD,** freelance writer and reviewer, Medscape, LLC. *Disclosure: Laurie Barclay, MD, has disclosed no relevant financial relationships.*

## Authors

*Disclosures:* **Aron J. Hall, DVM, MSPH; Valerie G. Eisenbart, DVM; Amy Lehman Etingüe, DVM; L. Hannah Gould, PhD; Ben A. Lopman, PhD;** *and* **Umesh D. Parashar, MBBS*,*** *have disclosed no relevant financial relationships.*
