# Supplementary material for: Epidemiology of Foodborne Norovirus Outbreaks, United States, 2001–2008
Source: Emerg Infect Dis. 2012 Oct;18(10):1566–73. doi: 10.3201/eid1810.120833 (PMC3471645; doi:10.3201/eid1810.120833)
Supplement: Related texts

# Research article: Epidemiology of Foodborne Norovirus Outbreaks, United States, 2001–2008

**DOI:** 10.3201/eid1810.120833

**Authors:** Aron J. Hall, Valerie G. Eisenbart, Amy Lehman Etingüe, L. Hannah Gould, Ben A. Lopman, Umesh D. Parashar

**Affiliations:** Centers for Disease Control and Prevention, Atlanta, Georgia, USA (A.J. Hall, V.G. Eisenbart, A. Lehman Etingüe, L.H. Gould, B.A. Lopman, U.D. Parashar);; University of Illinois, Urbana, Illinois, USA (V.G. Eisenbart);; and North Carolina State University, Raleigh, North Carolina, USA (A. Lehman Etingüe)

## Abstract

Noroviruses are the leading cause of foodborne illness in the United States. To better guide interventions, we analyzed 2,922 foodborne disease outbreaks for which norovirus was the suspected or confirmed cause, which had been reported to the Foodborne Disease Outbreak Surveillance System of the Centers for Disease Control and Prevention during 2001–2008. On average, 365 foodborne norovirus outbreaks were reported annually, resulting in an estimated 10,324 illnesses, 1,247 health care provider visits, 156 hospitalizations, and 1 death. In 364 outbreaks attributed to a single commodity, leafy vegetables (33%), fruits/nuts (16%), and mollusks (13%) were implicated most commonly. Infected food handlers were the source of 53% of outbreaks and may have contributed to 82% of outbreaks. Most foods were likely contaminated during preparation and service, except for mollusks, and occasionally, produce was contaminated during production and processing. Interventions to reduce the frequency of foodborne norovirus outbreaks should focus on food workers and production of produce and shellfish.

Noroviruses, the leading cause of foodborne illness in the United States, are responsible for an estimated 58% of all domestically acquired foodborne illness from known agents (*1*). The estimated 5.5 million annual foodborne norovirus illnesses in the United States, which constitute those associated with recognized outbreaks and those considered sporadic, result annually in 15,000 hospitalizations and 150 deaths and cost ≈$2 billion in health care expenses and lost productivity (*1*,*2*). Noroviruses are also the leading cause of foodborne disease outbreaks reported in the United States, accounting for about half of all foodborne outbreaks in which an etiologic agent is identified (*3*–*5*). Classified into the genus *Norovirus* within the family *Caliciviridae*, noroviruses are a genetically diverse group of nonenveloped, single-stranded RNA viruses, comprising at least 5 genogroups (GI–GV) and >35 genotypes (*6*). Since 2001, noroviruses within the GII.4 genotype have caused most viral gastroenteritis outbreaks worldwide (*7*).

Noroviruses can be spread through a variety of means, including direct person-to-person transmission through the fecal–oral route; ingestion of aerosolized vomitus; and indirect transmission through contaminated surfaces, food, or water. Norovirus has a low infectious inoculum (>18 viral particles) and is shed copiously by ill persons (10^5^–10^11^ viral copies per gram of feces), which enables its rapid and efficient spread (*8*–*10*). Noroviruses also remain infectious on surfaces for as long as 2 weeks and in water for >2 months (*11*,*12*) and are resistant to many common disinfectants (*13*,*14*). Foods can be contaminated with noroviruses at any point along the farm-to-fork continuum, although the most frequent pathways are thought to be through an infected food handler or exposure to water contaminated with fecal matter (e.g., surface water used for produce irrigation or water containing sewage discharge where shellfish grow) (*15*–*17*). Bivalve mollusks, such as oysters, bioaccumulate noroviruses in their body through filtration and selective binding mechanisms and therefore are readily contaminated when they are grown in harvesting areas contaminated with human feces (*18*).

Attribution of norovirus disease to specific foods and increasing understanding of the various contamination pathways that result in disease can help identify potential targets for interventions. Although most foodborne norovirus disease in the United States is not outbreak associated, outbreaks provide the most robust information about the foods that cause illness and the factors contributing to their contamination. The last published description of foodborne norovirus outbreaks in the United States was based on surveillance data from 1991 through 2000, before molecular diagnostic tools were widely available (*19*). Since that time, norovirus diagnostics have become incorporated more routinely into public health outbreak investigations, resulting in markedly increased recognition of norovirus illnesses and outbreaks. Using more recent surveillance data, we therefore sought to robustly describe foodborne norovirus outbreaks, including temporal, geographic, and demographic trends, and attribution to specific foods, settings, and contamination factors.

## Methods

### Data Source

Since 1973, the Centers for Disease Control and Prevention (CDC) has collected data on foodborne disease outbreaks from state, local, and territorial health departments through the Foodborne Disease Outbreak Surveillance System (FDOSS) (*20*). A foodborne disease outbreak is defined as >2 similar illnesses resulting from ingestion of a common food. Data collected for each outbreak include outbreak characteristics (e.g., dates, number of ill persons, locations, etiologic agents), case-patient characteristics (e.g., demographic characteristics, symptoms, health care seeking, and whether the illness resulted in death), setting of food preparation, contributing factors, and the implicated food vehicle(s) (*21*). Outbreaks of norovirus infection are considered laboratory confirmed if stool or vomitus specimens from >2 ill persons are positive for norovirus by reverse transcription PCR, enzyme immunoassay, or electron microscopy (*6*). Norovirus may be implicated as the suspected etiologic agent in the absence of laboratory confirmation when reasonable clinical or epidemiologic evidence exists, such as the previously validated Kaplan criteria (*22*). Data were extracted for all foodborne outbreaks in which norovirus was either a suspected or confirmed etiologic agent and the first illness occurred during 2001–2008.

### Data Analysis

Frequencies of outbreaks and outbreak-related illnesses, health care provider visits, hospitalizations, and deaths were calculated. Annual variations were analyzed by grouping data into seasonal years from July–June and comparing data with information from the 2 adjacent years. Seasonal trends and variations in reporting by state were also assessed. Because data on health care provider visits, hospitalizations, deaths, age groups, and sex were not always reported (i.e., they were provided for 63%, 66%, 69%, 82%, and 89% of all reported illnesses, respectively), the relative proportions of illnesses by age and outcome from the outbreaks that included such data were extrapolated to all reported outbreak-associated illnesses. For example, among the 68,452 outbreak-associated illnesses that included data on age, 22,301 (32.5%) occurred in persons >50 years of age; this proportion (32.5%) was applied to the total number of outbreak-associated illnesses reported (82,591) to yield the estimated number of outbreak-associated illnesses among those >50 years of age (26,872). Rates of reported outbreaks and outbreak-associated illnesses per 1,000,000 person-years were calculated by dividing the average annual number of these illnesses by the corresponding US intercensal estimate at the midpoint of the study period, July 2004 (*23*).

Food vehicles implicated in outbreaks were classified on the basis of a categorization hierarchy of 17 mutually exclusive commodity groups (*24*). If a food contained a single contaminated ingredient or all ingredients belonged to a single commodity, it was classified into that commodity. Food vehicles that contained ingredients from multiple commodities were classified as complex. Outbreaks were not attributed to any of the commodities if an implicated food vehicle could not be assigned to one of these commodities, multiple food vehicles were implicated, or no specific food vehicle was implicated.

Factors contributing to contamination and methods of food preparation were reported according to standard categorization schemes (*21*). Contributing factors were not mutually exclusive and were further grouped into the following categories: food handler contact, cross-contamination during preparation, contaminated raw product, and insufficient cooking and/or heating. Outbreak reports also indicated whether a food handler was specifically implicated as the source of contamination (e.g., handled the implicated food while symptomatic) rather than simply being a potential contributor. For implicated foods that could be classified into 1 of the 17 commodities, the likely point of contamination (POC) was assessed on the basis of a combination of contributing factors and whether a food handler was implicated as the source of contamination. POC was categorized to distinguish production or processing (i.e., a raw product contaminated from the environment or obtained from a polluted source) versus preparation or service (i.e., vehicle handled by infected food worker or cross-contamination during preparation). Those outbreaks with insufficient or conflicting information reported regarding the likely POC were classified as unknown.

The settings of food preparation in outbreaks were classified into the following categories: commercial (e.g., restaurant, grocery store, caterer), institutional (e.g., school, nursing home, prison), private (e.g., home, church, picnic), and other (including unknown); multiple settings could be selected in a given outbreak. Differences in median number of illnesses associated with outbreaks in different settings were assessed by using Wilcoxon rank-sum tests. χ^2^ tests were used to evaluate trends among categorical variables. Analyses were performed by using SAS v9.2 (SAS Institute Inc., Cary, NC, USA) and Epi Info v3.4.3 (CDC, Atlanta, GA, USA).

## Results

### Outbreak Characteristics

During 2001–2008, a total of 9,206 foodborne disease outbreaks were reported in the United States. For 2,922 (46%) of the 6,355 outbreaks with a known cause, norovirus was the confirmed or suspected cause, an average of 365 foodborne norovirus outbreaks annually (1.2 outbreaks/1,000,000 person-years). Of these, 1,683 (58%) outbreaks were laboratory confirmed, primarily on the basis of reverse transcription PCR. Genogroup information, reported in most confirmed norovirus outbreaks only since 2006, was available for 648 norovirus outbreaks, among which 111 (17%) were caused by genogroup I (GI) and 518 (80%) were caused by genogroup II (GII); 19 (3%) involved both GI and GII noroviruses. Sixteen (0.5%) norovirus outbreaks also involved other suspected or confirmed etiologic agents, including nontyphoidal *Salmonella* spp. (n = 4), adenovirus (n = 2), *Bacillus cereus* (n = 2), *Campylobacter* spp. (n = 2), *Clostridium perfringens* (n = 2), *Listeria monocytogenes* (n = 1), *Shigella* spp*.* (n = 1), and *Vibrio parahaemolyticus* (n = 1).

Foodborne norovirus outbreaks were reported from 49 states (all but Vermont) and the District of Columbia, with substantial state-to-state variation in the rate of reported outbreaks (Figure 1). The highest per capita rates of reported outbreaks were in Minnesota (6.2/1,000,000 person-years) and Oregon (6.1/1,000,000 person-years); the lowest reported rates were in Texas (0.25/1,000,000 person-years) and Kentucky (0.27/1,000,000 person-years). The greatest number of outbreaks was reported by California (n = 526, 18%). Residents from multiple states were affected in 138 (5%) outbreaks, of which 8 (6%) involved exposures to contaminated foods distributed to multiple states.

**Figure 1:** Total number and rate of reported foodborne norovirus outbreaks per 1,000,000 person-years by affected states, United States, 2001–2008. Number given in each state indicates the total number of outbreaks over the 8-year study period; shaded boxes in key indicate the reported rate by quartiles. Multistate outbreaks are assigned as outbreaks to each state involved.

Outbreaks were slightly more frequent in the winter months than in the rest of the year, with 957 (33%) outbreaks occurring during December–February; however, the seasonal pattern varied somewhat from year to year (Figure 2). The temporal pattern of outbreaks in which norovirus was confirmed by laboratory testing was similar to that of outbreaks in which norovirus was suspected as the etiologic agent. Although no consistent secular trend was observed over the 8-year study period, the number of outbreaks in 2006–07 (n = 442) was 24% higher than the average number of outbreaks during the 2 adjacent seasonal years of 2005–06 (n = 359) and 2007–08 (n = 352).

**Figure 2:** Number of reported foodborne norovirus outbreaks by month of first illness onset, United States, 2001–2008. Outbreaks are confirmed as caused by norovirus if fecal or vomitus specimens from >2 persons are positive for the virus by reverse transcription PCR, electron microscopy, or enzyme immunoassay.

On average, 10,324 reported illnesses were associated with foodborne norovirus outbreaks each year (Table 1). These included an estimated 1,247 (12%) health care provider visits, 156 (1.5%) hospitalizations, and 1 (0.01%) death annually. Most (80%) outbreak-associated illnesses affected adults >20 years of age, and 56% of illnesses affected women. Children <5 years of age had a significantly lower rate of foodborne norovirus outbreak–associated illness (11/1,000,000 person-years) than did all other age groups combined (37/1,000,000 person-years) (p<0.001). Among outbreaks for which data were available, the median incubation period was 33 hours (n = 2,348), and the median attack rate was 61% (n = 1,099).

**Table 1:** Estimated annual number and rate of reported illnesses associated with foodborne norovirus outbreaks by age, sex, and outcome, United States, 2001–2008

Characteristic	Estimated annual outbreak-associated illnesses*	
	No. (%)	Rate†
Age group, y		
<5	215 (2)	10.6
5–19	1,827 (18)	30.3
20–49	4,923 (48)	39.8
>50	3,359 (33)	37.7
Sex		
F	5,819 (56)	39.0
M	4,505 (44)	31.3
Outcome		
Health care provider visit	1,247 (12)	4.3
Hospitalization	156 (1.5)	0.5
Death	1 (0.01)	0.002
Total Illnesses	10,324 (100)	35.2

*Proportions of illnesses by age, sex, and outcomes among outbreaks for which such data were reported were extrapolated to all reported foodborne norovirus outbreak–associated illnesses. †Reported rate per 1,000,000 person-years calculated by dividing the number of illnesses by the corresponding US intercensal estimate at the study period midpoint (July 2004) (*23*).

Norovirus outbreaks involved foods prepared most often in commercial settings (83%) and less frequently in private (11%), institutional (8%), and other (12%) settings (Table 2). Outbreaks involving institutional settings were significantly larger in terms of total number of illnesses (median 36 illnesses/outbreak) than those in other settings (median 15 illnesses/outbreak) (p<0.001). The most commonly reported settings were restaurants or delicatessens (62%), caterers (11%), and private homes (10%).

**Table 2:** Settings of food preparation in foodborne norovirus outbreaks, United States, 2001–2008*

Setting†	No. (%) outbreaks	Median no. cases/outbreak (IQR)
Commercial	2,432 (83)	14 (7–26)
Restaurant or delicatessen	1,824 (62)	12 (6–23)
Caterer	313 (11)	25 (16–40)
Banquet facility	110 (4)	30 (19–59)
Grocery store	105 (4)	13 (9–18)
Commercial product served without further preparation	42 (1)	20 (11–30)
Wedding reception	22 (1)	25 (18–35)
Fair, festival, other temporary/mobile service	16 (1)	21 (6–50)
Institutional	248 (8)	36 (19–71)
School	91 (3)	43 (29–92)
Nursing home	66 (2)	35 (19–60)
Camp	29 (1)	34 (18–53)
Noncafeteria workplace	18 (1)	23 (13–56)
Prison or jail	15 (1)	58 (23–137)
Workplace cafeteria	12 (0.4)	22 (18–78)
Hospital	11 (0.4)	53 (16–72)
Day care center	4 (0.1)	31 (19–65)
Office setting	2 (0.1)	18 (15–21)
Private	336 (11)	16 (10–26)
Private home	296 (10)	15 (9–25)
Church, temple, or other place of worship	31 (1)	23 (12–40)
Picnic	9 (0.3)	19 (12–32)
Other/Unknown	267 (9)	28 (14–49)
All outbreaks	2,922 (100)	14 (8–30)

*IQR, interquartile range. †Multiple locations may be implicated in a given outbreak.

### Food Attribution

At least 1 food vehicle was implicated in 1,298 (44%) outbreaks, of which 534 (41%) involved a complex food, 364 (28%) involved a simple food that could be classified into one of the 17 commodities, 279 (21%) involved multiple foods, and 121 (9%) involved a single food item that could not be classified. The reasons for implicating specific foods included statistical evidence from epidemiologic investigations (55%); laboratory evidence, such as identification of the agent in the food (2%); compelling supportive information (30%); other data (0.1%); and prior experience in the absence of specific evidence (5%). For 8% of implicated foods, no reasons were given. Of the 813 outbreaks that involved complex or multiple foods, 328 (40%) implicated sandwiches, salads, or other foods eaten raw or lightly cooked.

Factors that may have contributed to contamination were provided in 886 (68%) of outbreaks with at least 1 implicated food; among these outbreaks, food handler contact with ready-to-eat food was identified in 725 (82%), consumption of a contaminated raw product in 111 (13%), cross-contamination during preparation in 109 (12%), and inadequate cooking or heating in 28 (3%). A food handler was specifically implicated as the source of contamination in 473 (53%) outbreaks. This determination was reportedly made on the basis of laboratory and epidemiologic evidence (33%), epidemiologic evidence only (45%), laboratory evidence only (4%), and prior experience only (17%); no reason for implicating a food handler was given for 2 of these outbreaks. No significant differences in contributing factors were identified between different settings where foods were prepared.

Among the 364 outbreaks with a single, simple food implicated, the most frequent commodities were leafy vegetables (33%), fruits/nuts (16%), and mollusks (13%), although all commodities except sprouts were implicated in at least 1 outbreak (Figure 3). Information was available to indicate the likely POC in 191 (52%) of these outbreaks, among which contamination during preparation or service was more frequent (85%, n = 162) than was contamination during production or processing (15%, n = 29) (p<0.001). All 26 mollusk-associated outbreaks in which the likely POC could be determined were caused by contamination during production or processing. Production or processing contamination was also indicated in 3 of the 109 outbreaks associated with either leafy greens or fruits/nuts in which a POC could be determined. Outbreaks involving all other commodities resulted either from contamination during preparation or service or from an unknown POC. A traceback was performed in 8 outbreaks, all of which involved mollusks; 3 of these resulted in a product recall.

**Figure 3:** Commodity and point of contamination implicated in reported norovirus outbreaks involving simple foods (consisting of a single commodity; n = 364), United States, 2001–2008. Point of contamination was classified as unknown if insufficient or conflicting information was provided in the outbreak report.

## Discussion

This study demonstrates the predominant etiologic role of norovirus in foodborne disease outbreaks in the United States, with an average of 1 foodborne norovirus outbreak reported every day, and highlights the frequency of norovirus contamination of raw and other ready-to-eat foods. Fresh produce, primarily leafy vegetables and fruits, was implicated in over half of all outbreaks that could be classified into a single commodity; ready-to-eat foods that contain fresh produce, such as sandwiches and salads, were frequently implicated complex vehicles. Mollusks, which are also commonly served raw or undercooked, were also implicated frequently. Food handler contact with raw and ready-to-eat foods was identified as the most common scenario resulting in foodborne norovirus outbreaks, underscoring the need to better understand and control endemic norovirus disease as a means of foodborne disease prevention. Contact with food handlers during preparation was cited in 82% of outbreaks as a possible contributor to contamination and was specifically implicated as the source of contamination in 53% of outbreaks. These proportions likely reflect lack of positive evidence and therefore may be underestimated because of disincentives for reporting illness and asymptomatic infections among food handlers (*25*,*26*). Most foods implicated in norovirus outbreaks were prepared in restaurants, delicatessens, and other commercial settings, which suggests that these are key locations for intervention. Steps to curtail contamination of ready-to-eat foods by food handlers in these settings include adherence to appropriate recommendations for hand washing and use of gloves; compliance with policies to prevent ill staff from working; and presence of a certified kitchen manager, as recommended by the Food Code of the US Food and Drug Administration (*27*).

With the exception of mollusk-associated outbreaks, we rarely identified contamination during production or processing in this analysis, although it is likely underrecognized in norovirus outbreaks. Indeed, the point of contamination could not be determined in ≈50% of single-commodity outbreaks, many of which may be additional instances of contamination before preparation. Oysters are well documented as food vehicles that are prone to norovirus contamination during production (*17*,*28*), but examples of contaminated produce have also been reported, including raspberries and lettuce, which are likely contaminated from irrigation waters (*16*,*29*). In a recent multicountry study, norovirus RNA was detected in 28%–50% of samples from leafy green vegetables and in 7%–34% of samples from soft red fruit (e.g., strawberries and raspberries) obtained from retail markets or directly from processing companies (*30*). These findings demonstrate that fresh produce often comes in contact with norovirus during production. However, because the available detection methods cannot distinguish infectious virus from noninfectious genomic material, the specific risks posed to public health from this potential contamination of produce remain unclear. Outbreak investigations can provide learning opportunities to possibly link such contamination with human illness through tracebacks and root-cause analyses to determine when, where, and how foods became contaminated. Unfortunately, no tracebacks were performed during produce-associated outbreaks included in this analysis, despite evidence in some of these outbreaks that suggested possible contamination before preparation.

No specific food vehicle was implicated in 56% of the foodborne norovirus outbreaks included in this analysis, a substantially higher proportion than that for foodborne outbreaks with bacterial etiologic agents (*3*–*5*). This discrepancy underscores the challenges in food attribution of norovirus outbreaks, given the potential for multiple transmission pathways, contamination of multiple vehicles by an ill food handler, and time lags in reporting by citizen complaint. However, the discrepancy may also reflect the relative deprioritization of investigating suspected norovirus outbreaks. Misperceptions may exist that foodborne norovirus outbreaks result only from local contamination events and afford little opportunity for further prevention and/or control. Such decisions regarding investigations are often made in the face of scant public health resources, inadequate staffing, and competing priorities (*31*). Overcoming these misperceptions, prioritizations, and limited resources are key challenges to improving the knowledge base for effective prevention and control of foodborne norovirus disease.

Although overall reporting of foodborne norovirus outbreaks has vastly improved since the 1990s (*19*), great disparity remains among states. A 25-fold difference in population-based rates of foodborne norovirus outbreaks was identified between the highest and lowest reporting states. Although these differences may be due in part to true variations in the incidence of foodborne norovirus outbreaks, they also suggest varying degrees of underreporting. Thus, rates of outbreaks and outbreak-associated illnesses captured through surveillance represent reporting rates, not true incidence, and likely underestimate the true incidence. Capacity of state and local health departments to investigate foodborne disease outbreaks varies widely, with the most notable limitations being lack of dedicated personnel and delayed notification of outbreaks (*31*). Efforts to better understand the gaps in foodborne outbreak response, including laboratory, epidemiologic, and environmental health capacity, may ultimately inform strategies to overcome the challenges of limited public health resources (*32*).

The increasing trend in the 1990s in the number of foodborne norovirus outbreaks reported appears to have leveled off, likely reflecting the availability of diagnostic testing for norovirus at almost all public health laboratories across US states. However, a substantial increase was observed during 2006–07, contemporaneous with the emergence of 2 new GII type 4 (GII.4) norovirus variants (*7*). Interestingly, the surge in foodborne outbreaks during the 2006–07 epidemic season appeared less pronounced than that observed among norovirus outbreaks in general (*7*,*33*), most of which result from direct person-to-person spread (*34*), as well sporadic norovirus-associated hospitalizations and deaths in the United States (*35*,*36*). Furthermore, foodborne norovirus outbreaks did not increase in association with emergence of a new GII.4 variant in the 2002–03 seasonal year, which has been previously correlated with other indicators of increased norovirus activity (*7*,*35*,*36*). These observations coupled with the paucity of genotype information reported directly through FDOSS underscores the need for and importance of CaliciNet, the recently implemented national laboratory surveillance network for norovirus outbreaks (*37*). Integration of sequence-based genotyping data with epidemiologic data will enable timely recognition of the impact of emergent noroviruses on foodborne disease and potentially identify links between outbreaks due to widely distributed food vehicles. Additionally, beginning in 2009, US data on enteric disease outbreaks associated with any mode of transmission, i.e., foodborne and nonfoodborne, are systematically collected through a single comprehensive system, the National Outbreak Reporting System (NORS), allowing for attribution of all norovirus outbreaks by mode of transmission and setting (*38*).

Despite the demonstrated prevalence and effects of these infections, relatively few evidence-based interventions exist for preventing and controlling foodborne norovirus disease. This study provides a comprehensive analysis of US foodborne norovirus outbreaks, highlighting both potential targets for interventions and key remaining data gaps. Fresh produce, mollusks, and ready-to-eat foods were identified as the primary vehicles of foodborne norovirus disease. Continued focus on food handlers in retail settings is warranted, although the role and mechanisms of contamination during production, particularly as they relate to fresh produce, remain unclear. To that end, further development and validation of analytic methods for the detection of norovirus in foods, particularly those that can quantitatively distinguish contamination levels correlating with infection risk and distinguish infectious from noninfectious virus, are critically needed (*32*). Likewise, there is ongoing need to define the duration of infectiousness of ill food handlers and to elucidate the role of asymptomatic food handlers in norovirus transmission, issues that have been hampered by the inability to culture human noroviruses in vitro. Other areas needing attention are improving understanding of the roles of various norovirus genotypes in different foods, identifying the environmental antecedents leading to food contamination, and the role of other transmission modes in propagating norovirus spread. Initiatives to improve outbreak surveillance and response can help address some of these data gaps (*35*,*37*–*39*), but only if public health agencies have adequate resources. Finally, as the development of candidate norovirus vaccines continues to progress (*40*), their possible public health utility in specific populations, such as food handlers, should be assessed.

## References

[1] Scallan E, Hoekstra RM, Angulo FJ, Tauxe RV, Widdowson M-A, Roy SL, Foodborne illness acquired in the United States—major pathogens. Emerg Infect Dis. 2011;17:7–15.2119284810.3201/eid1701.P11101PMC3375761

[2] Batz MB, Hoffmann S, Morris JG Jr. Ranking the risks: the 10 pathogen-food combinations with the greatest burden on public health: University of Florida; 2011 [cited 2012 Jul 26]. http://www.rwjf.org/files/research/72267report.pdf

[3] Centers for Disease Control and Prevention. Surveillance for foodborne disease outbreaks—United States, 2006. MMWR Morb Mortal Wkly Rep. 2009;58:609–15.19521332

[4] Centers for Disease Control and Prevention. Surveillance for foodborne disease outbreaks—United States, 2007. MMWR Morb Mortal Wkly Rep. 2010;59:973–9.20703204

[5] Centers for Disease Control and Prevention. Surveillance for foodborne disease outbreaks—United States, 2008. MMWR Morb Mortal Wkly Rep. 2011;60:1197–202.21900873

[6] Centers for Disease Control and Prevention. Updated norovirus outbreak management and disease prevention guidelines. MMWR Recomm Rep. 2011;60(RR-3):1–18.21368741

[7] Zheng DP, Widdowson MA, Glass RI, Vinje J. Molecular epidemiology of genogroup II–genotype 4 noroviruses in the United States between 1994 and 2006. J Clin Microbiol. 2010;48:168–77. 10.1128/JCM.01622-0919864482PMC2812295

[8] Teunis PF, Moe CL, Liu P, Miller SE, Lindesmith L, Baric RS, Norwalk virus: how infectious is it? J Med Virol. 2008;80:1468–76. 10.1002/jmv.2123718551613

[9] Atmar RL, Opekun AR, Gilger MA, Estes MK, Crawford SE, Neill FH, Norwalk virus shedding after experimental human infection. Emerg Infect Dis. 2008;14:1553–7. 10.3201/eid1410.08011718826818PMC2609865

[10] Aoki Y, Suto A, Mizuta K, Ahiko T, Osaka K, Matsuzaki Y. Duration of norovirus excretion and the longitudinal course of viral load in norovirus-infected elderly patients. J Hosp Infect. 2010;75:42–6. 10.1016/j.jhin.2009.12.01620304524

[11] Cheesbrough JS, Barkess-Jones L, Brown DW. Possible prolonged environmental survival of small round structured viruses. J Hosp Infect. 1997;35:325–6. 10.1016/S0195-6701(97)90230-99152829

[12] Seitz SR, Leon JS, Schwab KJ, Lyon GM, Dowd M, McDaniels M, Norovirus infectivity in humans and persistence in water. Appl Environ Microbiol. 2011;77:6884–8. 10.1128/AEM.05806-1121856841PMC3187119

[13] Park GW, Barclay L, Macinga D, Charbonneau D, Pettigrew CA, Vinje J. Comparative efficacy of seven hand sanitizers against murine norovirus, feline calicivirus and GII.4 norovirus. J Food Prot. 2010;73:2232–8.2121974110.4315/0362-028x-73.12.2232

[14] Park GW, Sobsey MD. Simultaneous comparison of murine norovirus, feline calicivirus, coliphage MS2, and GII.4 norovirus to evaluate the efficacy of sodium hypochlorite against human norovirus on a fecally soiled stainless steel surface. Foodborne Pathog Dis. 2011;8:1005–10. 10.1089/fpd.2010.078221457050

[15] Lynch M, Painter J, Woodruff R, Braden C. Surveillance for foodborne-disease outbreaks—United States, 1998–2002. MMWR Surveill Summ. 2006;55:1–42.17093388

[16] Falkenhorst G, Krusell L, Lisby M, Madsen SB, Bottiger B, Molbak K. Imported frozen raspberries cause a series of norovirus outbreaks in Denmark, 2005. Euro Surveill. 2005;10:E050922.2.1678823510.2807/esw.10.38.02795-en

[17] Westrell T, Dusch V, Ethelberg S, Harris J, Hjertqvist M, Jourdan-da Silva N, Norovirus outbreaks linked to oyster consumption in the United Kingdom, Norway, France, Sweden and Denmark, 2010. Euro Surveill. 2010;15:19524.20350499

[18] Maalouf H, Schaeffer J, Parnaudeau S, Le Pendu J, Atmar RL, Crawford SE, Strain-dependent norovirus bioaccumulation in oysters. Appl Environ Microbiol. 2011;77:3189–96. 10.1128/AEM.03010-1021441327PMC3126434

[19] Widdowson MA, Sulka A, Bulens SN, Beard RS, Chaves SS, Hammond R, Norovirus and foodborne disease, United States, 1991–2000. Emerg Infect Dis. 2005;11:95–102. 10.3201/eid1101.04042615705329PMC3294339

[20] Centers for Disease Control and Prevention. Foodborne disease outbreak surveillance. September 2011 [cited 2012 Jul 13]. http://www.cdc.gov/outbreaknet/surveillance_data.html

[21] Centers for Disease Control and Prevention. Electronic Foodborne Outbreak Reporting System: investigation of a foodborne outbreak (CDC form 52.13). November 2004 [cited 2012 Feb 22]. http://www.cdc.gov/outbreaknet/toolkit/efors_form.pdf

[22] Turcios RM, Widdowson MA, Sulka AC, Mead PS, Glass RI. Reevaluation of epidemiological criteria for identifying outbreaks of acute gastroenteritis due to norovirus: United States, 1998–2000. Clin Infect Dis. 2006;42:964–9. 10.1086/50094016511760

[23] National Center for Health Statistics. Postcensal estimates of the resident population of the United States for July 1, 2000–July 1, 2009, by year, county, age, bridged race, Hispanic origin, and sex (Vintage 2009). June 20, 2010 [cited 2011 May 11]. http://www.cdc.gov/nchs/nvss/bridged_race.htm

[24] Painter JA, Ayers T, Woodruff R, Blanton E, Perez N, Hoekstra RM, Recipes for foodborne outbreaks: a scheme for categorizing and grouping implicated foods. Foodborne Pathog Dis. 2009;6:1259–64. 10.1089/fpd.2009.035019968563

[25] Phillips G, Tam CC, Rodrigues LC, Lopman B. Prevalence and characteristics of asymptomatic norovirus infection in the community in England. Epidemiol Infect. 2010;138:1454–8. 10.1017/S095026881000043920196905

[26] Todd EC, Greig JD, Bartleson CA, Michaels BS. Outbreaks where food workers have been implicated in the spread of foodborne disease. Part 5. Sources of contamination and pathogen excretion from infected persons. J Food Prot. 2008;71:2582–95.1924491910.4315/0362-028x-71.12.2582

[27] US Food and Drug Administration. Food code, 2005 [cited 2012 Jul 26]. http://www.fda.gov/Food/FoodSafety/RetailFoodProtection/FoodCode/FoodCode2009/default.htm

[28] Le Guyader FS, Atmar RL, Le Pendu J. Transmission of viruses through shellfish: when specific ligands come into play. Curr Opin Virol. 2012;2:103–10. 10.1016/j.coviro.2011.10.02922440973PMC3839110

[29] Ethelberg S, Lisby M, Bottiger B, Schultz AC, Villif A, Jensen T, Outbreaks of gastroenteritis linked to lettuce, Denmark, January 2010. Euro Surveill. 2010;15:19484.20158982

[30] Baert L, Mattison K, Loisy-Hamon F, Harlow J, Martyres A, Lebeau B, Review: norovirus prevalence in Belgian, Canadian and French fresh produce: a threat to human health? Int J Food Microbiol. 2011;151:261–9. 10.1016/j.ijfoodmicro.2011.09.01322014587

[31] Centers for Disease Control and Prevention. Food safety epidemiology capacity in state health departments—United States, 2010. MMWR Morb Mortal Wkly Rep. 2011;60:1701–4.22189892

[32] Biggerstaff GK, Wright JG, Mitchell JR. FoodCORE: foodborne diseases centers for outbreak response enhancement—improving foodborne disease outbreak response capacity in state and local health departments. In: 2012 International Conference on Emerging Infectious Diseases program and abstracts book, March 11–14, Atlanta, Georgia, USA [cited 2012 Jul 26]. http://www.iceid.org/images/iceid_2012_finalprogram_final.pdf

[33] Centers for Disease Control and Prevention. Norovirus activity—United States, 2006–2007. MMWR Morb Mortal Wkly Rep. 2007;56:842–6.17717513

[34] Yen C, Wikswo ME, Lopman BA, Vinje J, Parashar UD, Hall AJ. Impact of an emergent norovirus variant in 2009 on norovirus outbreak activity in the United States. Clin Infect Dis. 2011;53:568–71. 10.1093/cid/cir47821832262

[35] Lopman BA, Hall AJ, Curns AT, Parashar UD. Increasing rates of gastroenteritis hospital discharges in US adults and the contribution of norovirus, 1996–2007. Clin Infect Dis. 2011;52:466–74. 10.1093/cid/ciq16321258098

[36] Hall AJ, Curns AT, McDonald LC, Parashar UD, Lopman BA. The roles of *Clostridium difficile* and norovirus among gastroenteritis-associated deaths in the United States, 1999–2007. Clin Infect Dis. 2012;55:216–23. 10.1093/cid/cis38622491338

[37] Vega E, Barclay L, Gregoricus N, Williams K, Lee D, Vinje J. Novel surveillance network for norovirus gastroenteritis outbreaks, United States. Emerg Infect Dis. 2011;17:1389–95.2180161410.3201/eid1708.101837PMC3381557

[38] Manikonda KL, Wikswo ME, Roberts VA, Richardson L, Gould LH, Yoder JS, The national outbreak reporting system: preliminary results of the first year of surveillance for multiple modes of transmission—United States, 2009. In: 2012 International Conference on Emerging Infectious Diseases program and abstracts book, March 11–14, Atlanta, Georgia, USA [cited 2012 Jul 26]. http://www.iceid.org/images/iceid_2012_finalprogram_final.pdf

[39] Centers for Disease Control and Prevention. National Voluntary Environmental Assessment Information System (NVEAIS). December 2011 [cited 2012 April 3]. http://www.cdc.gov/nceh/ehs/EHSNet/resources/nveais.htm

[40] Atmar RL, Bernstein DI, Harro CD, Al-Ibrahim MS, Chen WH, Ferreira J, Norovirus vaccine against experimental human Norwalk virus illness. N Engl J Med. 2011;365:2178–87. 10.1056/NEJMoa110124522150036PMC3761795

# Research article: Epidemiology of Foodborne Norovirus Outbreaks, United States, 2001–2008

**DOI:** 10.3201/eid1810.120833

**Authors:** Aron J. Hall, Valerie G. Eisenbart, Amy Lehman Etingüe, L. Hannah Gould, Ben A. Lopman, Umesh D. Parashar

**Affiliations:** Centers for Disease Control and Prevention, Atlanta, Georgia, USA (A.J. Hall, V.G. Eisenbart, A. Lehman Etingüe, L.H. Gould, B.A. Lopman, U.D. Parashar);; University of Illinois, Urbana, Illinois, USA (V.G. Eisenbart);; and North Carolina State University, Raleigh, North Carolina, USA (A. Lehman Etingüe)

## Medscape CME Quiz

## Earning CME Credit

To obtain credit, you should first read the journal article. After reading the article, you should be able to answer the following, related, multiple-choice questions. To complete the questions (with a minimum 70% passing score) and earn continuing medical education (CME) credit, please go to www.medscape.org/journal/eid. Credit cannot be obtained for tests completed on paper, although you may use the worksheet below to keep a record of your answers. You must be a registered user on Medscape.org. If you are not registered on Medscape.org, please click on the New Users: Free Registration link on the left hand side of the website to register. Only one answer is correct for each question. Once you successfully answer all post-test questions you will be able to view and/or print your certificate. For questions regarding the content of this activity, contact the accredited provider, CME@medscape.net. For technical assistance, contact CME@webmd.net. American Medical Association’s Physician’s Recognition Award (AMA PRA) credits are accepted in the US as evidence of participation in CME activities. For further information on this award, please refer to http://www.ama-assn.org/ama/pub/category/2922.html. The AMA has determined that physicians not licensed in the US who participate in this CME activity are eligible for *AMA PRA Category 1 Credits*™. Through agreements that the AMA has made with agencies in some countries, AMA PRA credit may be acceptable as evidence of participation in CME activities. If you are not licensed in the US, please complete the questions online, print the certificate and present it to your national medical association for review.

## Article Title: Epidemiology of Foodborne Norovirus Outbreaks, United States, 2001–2008

## CME Questions

**1. You are an infectious disease expert consulting to a US public health office regarding prevention and reducing the impact of norovirus outbreaks. Based on the study by Dr. Hall and colleagues, which of the following statements about general characteristics and outcomes of US foodborne norovirus outbreaks during 2001-2008 is most likely to appear in your report?**

A. On average, about 1 norovirus outbreak occurred every week

B. The increasing trend that began in the 1990s in the number of reported foodborne norovirus outbreaks continued through 2008

C. Norovirus outbreaks have been linked to an estimated average number of 10,324 illnesses, 1,247 healthcare provider visits, and 156 hospitalizations each year

D. No deaths have been attributed to norovirus

**2. Based on the study by Dr. Hall and colleagues, which of the following statements about sources of US norovirus outbreaks is most likely correct?**

A. Ground meat was often implicated

B. The most common single source was shellfish

C. Most foods were likely contaminated during production and processing

D. Contact with food handlers during preparation was cited in 82% of outbreaks as a possible contributor to contamination

**3. Based on the study by Dr. Hall and colleagues, which of the following statements about recommended interventions to reduce the frequency and impacts of foodborne norovirus outbreaks would most likely be correct?**

A. Food shipping plants are the best target for intervention

B. Food handlers preparing ready-to-eat foods should adhere to hand washing and gloving recommendations and to ill worker exclusion policies

C. A certified kitchen manager is unnecessary in most delis and restaurants

D. Analytic methods to detect norovirus in foods are well established

## Activity Evaluation

**Table:** 

**1. The activity supported the learning objectives.**				
Strongly Disagree				Strongly Agree
1	2	3	4	5
**2. The material was organized clearly for learning to occur.**				
Strongly Disagree				Strongly Agree
1	2	3	4	5
**3. The content learned from this activity will impact my practice.**				
Strongly Disagree				Strongly Agree
1	2	3	4	5
**4. The activity was presented objectively and free of commercial bias.**				
Strongly Disagree				Strongly Agree
1	2	3	4	5
